# Development and evaluation of a deep learning system for screening real-world multiple abnormal findings based on ultra-widefield fundus images

**DOI:** 10.3389/fmed.2025.1584378

**Published:** 2025-06-03

**Authors:** Haodong Xiao, Lie Ju, Zupeng Lu, Shiguang Zhang, Yan Jiang, Yuan Yang, Xuerui Zhang, Wenting Zhang, Huanyu Liu, Tingyi Liang, Jianing Ren, Jiawei Yin, Xiaoyu Liu, Tong Ma, Lin Wang, Wei Feng, Kaimin Song, Yuzhong Chen, Zongyuan Ge, Qian Shao, Jie Peng, Jili Chen, Peiquan Zhao

**Affiliations:** ^1^Department of Ophthalmology, Xinhua Hospital Affiliated to Shanghai Jiao Tong University School of Medicine, Shanghai, China; ^2^Beijing Airdoc Technology Co., Ltd., Beijing, China; ^3^Monash Medical AI Group, Monash University, Clayton, VIC, Australia; ^4^Department of Ophthalmology, Shanghai Children’s Hospital, School of Medicine, Shanghai Jiao Tong University, Shanghai, China; ^5^Zhenjiang Ruikang Hospital, Zhenjiang, China; ^6^Shibei Hospital, Shanghai, China; ^7^Department of Ophthalmology, Yichang Central People’s Hospital, The First College of Clinical Medical Science, China Three Gorges University, Yichang, China

**Keywords:** deep learning, ultra-widefield fundus images, real-world images, artifact removal, multiple abnormal findings

## Abstract

**Purpose:**

To develop and evaluate a deep learning system for screening multiple abnormal findings including hemorrhages, drusen, hard exudates, cotton wool spots and retinal breaks using ultra-widefield fundus images.

**Methods:**

The system consisted of three modules: (I) quality assessment module, (II) artifact removal module and (III) lesion recognition module. In Module III, a heatmap was generated to highlight the lesion area. A total of 4,521 UWF images were used for the training and internal validation of the DL system. The system was evaluated in two external validation datasets consisting of 344 images and 894 images from two other hospitals. The performance of the system in these two datasets was compared with or without Module II.

**Results:**

In both external validation datasets, the deep learning system made better performance when recognizing lesions on processed images after Module II than on original images without Module II. Module II-enhanced preprocessing improved Module III’s five-lesion recognition performance by an average of 6.73% and 14.4% areas under the curves, 14.47% and 19.62% accuracy in the two external validations.

**Conclusion:**

Our system showed reliable performance for detecting MAF in real-world UWF images. For deep learning systems to recognize real-world images, the artifact removal module was indeed helpful.

## Introduction

Retinal diseases have become one of the leading causes of irreversible vision loss and blindness worldwide (1). For example, diabetic retinopathy (DR) is the most common vision-threatening fundus disease in working population (2). Age-related macular degeneration (AMD) is one of the leading causes of blindness in elder population (3). Rhegmatogenous retinal detachment can cause vision loss in any age, especially in myopic population (4), which is preventable when retinal breaks were timely found and treated. Typically, ophthalmologists make diagnoses of these vision-threatening diseases by detection of certain clinical signs, such as retinal hemorrhage, drusen, hard exudate, cotton wool spots and retinal breaks. Early detection of the former retinal lesions contributes to timely management and better prognosis. However, the lack of retinal specialists, particularly in underserved regions, impedes timely diagnosis, exacerbating the burden on patients and healthcare systems.

AI has achieved impressive results in detecting single retinal diseases using UWF imaging and deep learning (5–11). However, these systems struggle with real-world clinical challenges (12). They cannot identify multiple coexisting lesions often seen in practice. They also depend heavily on pre-processed, high-quality images from eyes with clear media. Some studies report good performance in multi-lesion detection (13–18), but their clinical value is limited. These models use retrospective data with artificially high disease rates and strict quality control, making them poorly representative of actual clinical settings (19–21). This creates an urgent need for practical AI systems that can reliably detect multiple lesions in raw, diverse patient images.

The aim of this article was to develop and evaluate a deep learning system for screening and displaying lesion heatmaps of multiple abnormal findings (hemorrhages, drusen, hard exudates, cotton wool spots and retinal breaks) based on ultra-widefield fundus images and to verify its performance on an internal validation dataset and two independent external validation datasets.

## Materials and methods

### Data collection

For dataset establishment, initial random 4,521 UWF images were obtained from patients presenting for fundus evaluation between November 2017 and January 2019 at Xinhua Hospital (XHH) affiliated to Shanghai Jiao Tong University School of Medicine. A total of 344 high-quality UWF images were selected from patients examined in (ZRH) in 2022 and 894 high-quality UWF images were selected from patients examined in Shibei Hospital (SBH) Jingan District Shanghai in 2024, both for independent external validation. All images were captured using ultra-wide filed Scanning Laser Ophthalmoscopy (OPTOS Daytona P200T, Dunfermline, United Kingdom) with 200° fields of view in one shot. Patients underwent this examination without mydriasis. All images were checked for duplicate acquisition and de-identified prior to transfer to study investigators. The study was approved by the Ethics Committees of Xinhua Hospital Affiliated to Shanghai Jiao Tong University School of Medicine, Zhenjiang Ruikang Hospital, and Shibei Hospital. And this study followed the tenets of the Declaration of Helsinki.

### Characteristics of the datasets

A total of 4,521 UWF images varying in quality from 1,504 subjects examined in Xinhua Hospital were included in this study for algorithm development and internal validation. This development dataset was used for training and internal validation. The external validation dataset consisting of 344 high-quality UWF images was collected from 243 patients examined in Zhenjiang Ruikang Hospital in 2022. And the other external validation dataset consisting of 894 high-quality UWF images was collected from 500 patients from Shibei Hospital in 2024. The demographics and image characteristics of XHH dataset, ZRH dataset and SBH dataset are summarized in Supplementary Table 1. Noted that a UWF image may contain more than one lesion.

### Image labeling and reference standard

Training a deep learning system requires a reliable reference standard. Our system consisted of three modules: (I) quality assessment module, (II) artifact removal module and (III) lesion recognition module. For Module I training, we randomly extracted 500 of 4,521 UWF images from XHH dataset as the training set. The quality of the UWF images was first assessed by two board-certified retinal specialists with more than 5 years of clinical experience. The initially collected images were categorized as high, acceptable or low quality based on the clarity of the fundus structure and the visible range. Additional 50 images from each quality category are selected for internal validation on Module I. After training and testing Module I, only 4,289 high-quality images were kept for further development. We randomly selected 300 high-quality images as the training set for Module II. Artifacts such as patients’ eyelids or the examiner’s finger in these images were labeled (if present) by two board-certified retinal specialists with over 5 years of clinical experience. The results were used as a reference standard for the training of Module II. To evaluate the performance of Module II, we conducted additional analyses by training Module II with different sizes of training data and evaluating their effect on Module III’s final performance.

After artifacts’ removal in Module II, fully-pre-processed 4,289 UWF images were prepared for lesion recognition in Module III. And 3,789 images were randomly selected for training with 500 images left for internal validation for tuning and early stopping. Since we aimed to compare the performance of the DL system with and without Module II, we recruited five board-certified retinal specialists to perform lesion classification on all high-quality UWF images after Module II and those only after Module I. They annotated the observed abnormal findings (hemorrhages, drusen, hard exudates, cotton wool spots and retinal breaks) and their corresponding lesion location information according to eight non-overlapping regions (macular, temporal, superior temporal, inferior temporal, superior nasal, inferior nasal, superior disk and inferior disk areas) using LabelMe software. To ensure accurate identification of target lesions, all anonymous images were graded independently by the five retinal specialists. Only when there was agreement between the five retinal ophthalmologists would a consensus identification be made. Any level of disagreement resulted in a discussion between the retinal ophthalmologists. Disputed images were adjudicated by another senior retinal specialist with over 30 years of experience. The results of the annotation were also used as a reference standard for the internal and external validation datasets. The data splitting was performed at the patient level so images from the same patient were assigned either to the training set or to the validation set.

### Development of the deep learning classification model

**FIGURE 1:**
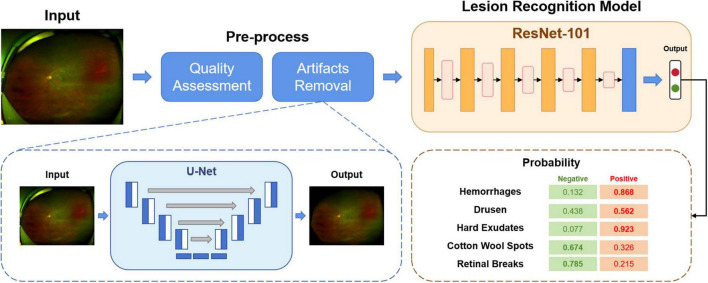
The overview of our proposed deep learning-based screening system.

As shown in Figure 1, the overall diagnostic framework consists of three modules: (I) quality assessment module; (II) artifact removal module; (III) lesion recognition module.

In Module I, as explained above, all the images initially collected were categorized into high-quality, acceptable or low-quality. The aim of this module is to automatically select the available images for training or testing. The images and their corresponding quality annotations were used to train a classification network. The model produces the predictions of the quality of the input images. In this study, only high-quality images were kept for further use.

Module II aims to remove artifacts from the UWF fundus images, such as eyelids. The presence of artifacts is a significant hurdle for UWF fundus images. By removing the irrelevant regions, the deep learning model can learn more specific and valuable information. A segmentation network was trained to predict whether a pixel was an artifact or a fundus area. For detected artifact pixels, we replaced them with black pixels with the value (RGB: 0, 0, 0). After Module I and Module II, the original images were pre-processed, ready for Module III training.

In Module III, a unified classification network was trained to diagnose different lesions. This module outputs the probability of the inputs being negative or positive for a particular lesion, such as hemorrhages, drusen or retinal breaks.

### Implementation

#### Image preprocessing and augmentation

All images were uniformly resized to 512 × 512 pixels before being used in different modules. In order to speed up learning and to achieve faster convergence, the value pixels were normalized from (0, 255) to (0, 1) for all channels. To increase the number of images available for training and to avoid overfitting, we applied data augmentation techniques during the training phase with random vertical/horizontal flip with probability 0.5, random rotation up to 90°, and brightness shift in the range 0.8–1.6.

#### Deep learning network

For Module I, we use the MobileNetV3 (22) as the backbone. The task of assessing the quality of UWF fundus images can be much easier than other diagnostic tasks. MobileNet is a lightweight deep neural network that combines precision and efficiency. It is flexible enough to be embedded in many mobile devices with low resource cost.

In Module II, a semantic segmentation network U-Net (23) was trained to detect the regions of artifacts as pre-processing.

Followed by Module I and II, a classification model built by ResNet-101 (24) was used in Module III for abnormality detection. ResNet is a widely used CNN architecture that addresses the “vanishing gradient” problem.

More technically, for Module I and Module III, the Adam (25) optimizer was used for backpropagation to minimize the object loss functions (cross-entropy loss). The learning rate was set to 3e-4. For Module II, we use the Adadelta (26) optimizer to update the parameters of the U-net. We have the following settings: learning rate = 1.0, rho = 0.95, epsilon = none and decay = 0. For Module I and Module III, the batch size was set to 128, while for Module II, the batch size was set to 16. Each module was trained for 50 epochs, and 5-fold cross-validation was performed to evaluate the robustness of the models and report the average results on external datasets with 95% CI. Early stopping was applied if the validation loss did not decrease for 10 consecutive epochs.

The experimental environment was built using Ubuntu version 18.04.4 LTS 64-bit with GPU RTX 3090 and 24 GB of memory. The deep neural network implementation was based on PyTorch platform version 1.8.1 and CUDA version 11.2.

### Visualization of image features

To highlight which regions contribute most to the diagnostic results of the model, we used gradient-weighted class activation mapping (Grad-CAM) (27) to visualize the image features. Grad-CAM is a training-free technique that uses the gradients that flow into the final convolutional layer to produce a coarse localization map that highlights the important regions in the image for predicting the specific category. Errors were also inevitable when the DL system did the classification work. We reviewed all misclassified images to determine the reasons for the misclassification. And for all true-positive and false-positive images, heatmaps highlighted any abnormal findings, including hemorrhages, drusen, hard exudates, cotton wool spots and/or retinal breaks. Image pixels with a higher impact on the model’s prediction have a heatmap color closer to the red spectrum in the jet color map, while those with a lower impact have a color closer to the blue spectrum.

### Statistical analysis

The performance of our DL system was assessed using accuracy, sensitivity and specificity that yielded the highest harmonic mean with the 95% confidence intervals. In addition, a receiver operating characteristic (ROC) curves and areas under the curves (AUC) of ROC for each abnormal finding was drawn. To determine the optimal classification thresholds for each lesion type, we employed the Youden Index, which maximizes the sum of sensitivity and specificity. Other metrics were used for more views of the results: Sensitivity = TP/(TP + FN), Specificity = TN/(TN + FP), Precision = TP/(TP + FP), Accuracy = (TP + TN)/(TP + TN + FP + FN), and F1 Score = 2 * Precision * Sensitivity/(Precision + Sensitivity), where TP is truly positive, TN is true negative, FP is false positive, and FN is false negative. 95% confidence interval was also applied and presented to assess the DL system. All statistical analyses were performed using Python 3.7.3 (Wilmington, Delaware, United States).

## Results

### Evaluation of module I and module II

**FIGURE 2:**
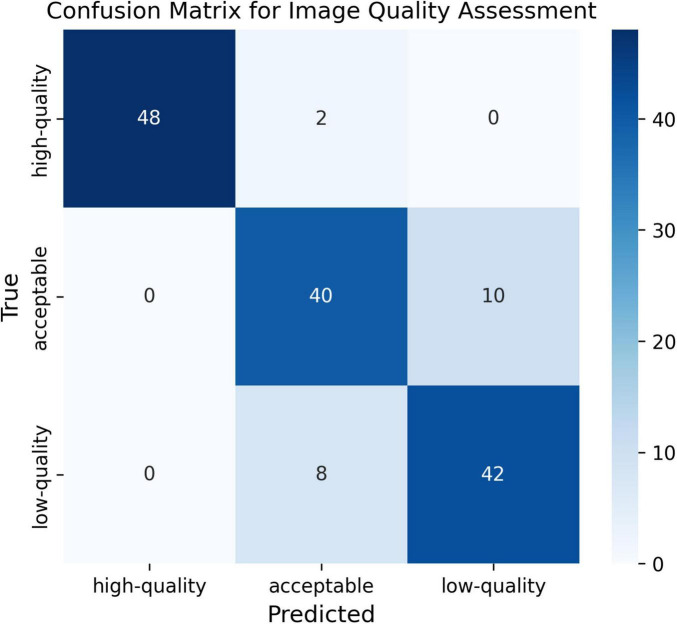
Confusion matrix for image quality assessment.

To quantitatively evaluate the trained Module I, a confusion matrix was plotted using 50 samples selected for each category. As seen in the confusion matrix (Figure 2), although there are samples that are difficult to distinguish between the “acceptable” and “low-quality” categories, we ultimately selected only the “high-quality” samples for the training of subsequent modules. This demonstrates that Module I has a strong discriminative ability in quality assessment.

**FIGURE 3:**
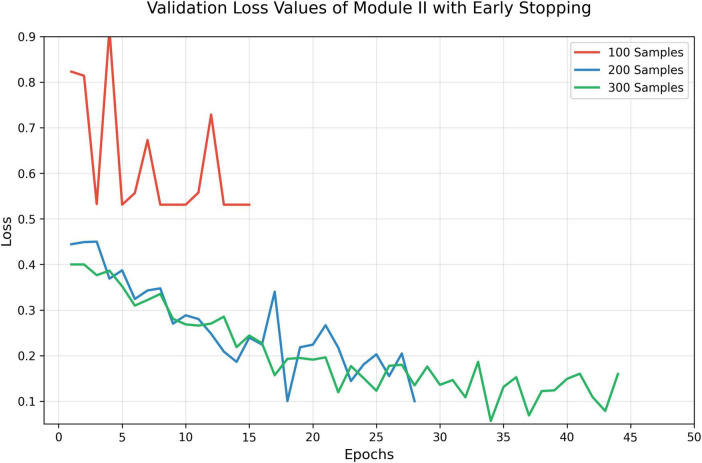
Validation loss with early stopping.

To preliminarily evaluate the performance of trained Module II, early stopping approach was used to train Module II with different sizes of training data (Figure 3). Interestingly, we found that even a small number of annotated images were sufficient to bring noticeable improvements, suggesting a favorable cost-benefit ratio for Module II development.

### Evaluation of screening models on different abnormalities

**FIGURE 4:**
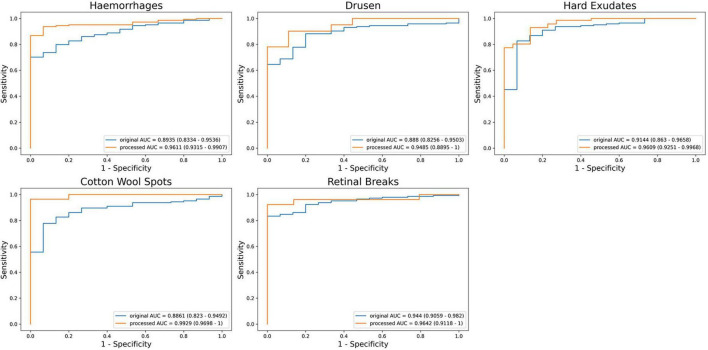
The receiver operating characteristic (ROC) of MAF on Zhenjiang Ruikang Hospital (ZRH) external validation dataset.

To evaluate the effectiveness of artifact removal module (Module II) and screening models in two external validation datasets, we presented the results on UWF images wo/w artifacts removed, which were denoted as “Original” and “Processed,” respectively. The results of ZRH dataset were shown in Table 1 and Figure 4. With Module II, the system demonstrated improved performance in AUC, sensitivity, and accuracy for recognizing all five abnormalities. And the results for SBH dataset are presented in Table 2. Similarly, in the presence of Module II, our system exhibited better performance in AUC, sensitivity, and accuracy for recognizing all five abnormalities.

**TABLE 1:** Performance of the screening models on MAF on Zhenjiang Ruikang Hospital (ZRH) external validation dataset without Module II —-(Original) and with Module II (Processed), respectively.

Original	Hemorrhages	Drusen	Hard exudates	Cotton wool spots	Retinal breaks
AUC	0.8935 (0.8334–0.9536)	0.8880 (0.8256–0.9503)	0.9144 (0.8630–0.9658)	0.8861 (0.8230–0.9492)	0.9440 (0.9059–0.9820)
Sensitivity	0.6944 (0.5713–0.8176)	0.8750 (0.8075–0.9425)	0.8194 (0.7318–0.9071)	0.7708 (0.6679–0.8738)	0.8264 (0.7411–0.9116)
Specificity	1.0000 (1.0000–1.0000)	0.8000 (0.7060–0.8940)	0.9333 (0.8903–0.9763)	0.9333 (0.8903–0.9763)	1.0000 (1.0000–1.0000)
Precision	1.0000 (1.0000–1.0000)	0.9767 (0.9555–0.9980)	0.9916 (0.9799–1.0000)	0.9911 (0.9790–1.0000)	1.0000 (1.0000–1.0000)
Accuracy	0.7233 (0.6072–0.8394)	0.8679 (0.7977–0.9381)	0.8302 (0.7462–0.9141)	0.7862 (0.6878–0.8845)	0.8428 (0.7632–0.9223)
F1 score	0.8197 (0.7321–0.9072)	0.9231 (0.8755–0.9707)	0.8973 (0.8388–0.9559)	0.8672 (0.7967–0.9377)	0.9049 (0.8495–0.9603)
**Processed**	**Hemorrhages**	**Drusen**	**Hard exudates**	**Cotton wool spots**	**Retinal breaks**
AUC	0.9611 (0.9315–0.9907)	0.9485 (0.8895–1.0000)	0.9609 (0.9251–0.9968)	0.9929 (0.9698–1.0000)	0.9642 (0.9118–1.0000)
Sensitivity	0.9306 (0.8863–0.9748)	0.8780 (0.7774–0.9787)	0.9155 (0.8595–0.9715)	0.9286 (0.8525–1.0000)	0.8846 (0.7913–0.9779)
Specificity	0.9333 (0.8903–0.9763)	0.8889 (0.7940–0.9838)	0.8636 (0.7892–0.9381)	1.0000 (1.0000–1.0000)	1.0000 (1.0000–1.0000)
Precision	0.9926 (0.9817–1.0000)	0.9730 (0.9325–1.0000)	0.9559 (0.9175–0.9943)	1.0000 (1.0000–1.0000)	1.0000 (1.0000–1.0000)
Accuracy	0.9308 (0.8867–0.9749)	0.8800 (0.7804–0.9796)	0.9032 (0.8425–0.9639)	0.9535 (0.8930–1.0000)	0.9455 (0.8808–1.0000)
F1 score	0.9606 (0.9307–0.9905)	0.9231 (0.8476–0.9985)	0.9353 (0.8874–0.9832)	0.9630 (0.9093–1.0000)	0.9388 (0.8703–1.0000)

Values are the highest harmonic mean with the 95% confidence intervals.

**TABLE 2:** Performance of the screening models on MAF on Shibei Hospital (SBH) external validation dataset without Module II (Original) and with Module II (Processed), respectively.

Original	Hemorrhages	Drusen	Hard exudates	Cotton wool spots	Retinal breaks
AUC	0.8312 (0.7715–0.8907)	0.8145 (0.7532–0.8738)	0.8524 (0.7921–0.9128)	0.8043 (0.7456–0.8629)	0.8739 (0.8342–0.9136)
Sensitivity	0.6427 (0.5231–0.7623)	0.8342 (0.7629–0.9055)	0.7736 (0.6824–0.8648)	0.7259 (0.6253–0.8265)	0.7824 (0.6935–0.8713)
Specificity	0.9718 (0.9234–1.0000)	0.7538 (0.6642–0.8434)	0.8925 (0.8417–0.9433)	0.8934 (0.8432–0.9436)	0.9741 (0.9248–1.0000)
Precision	0.9735 (0.9246–1.0000)	0.9437 (0.9123–0.9751)	0.9648 (0.9229–1.0000)	0.9654 (0.9241–1.0000)	1.0000 (1.0000–1.0000)
Accuracy	0.6834 (0.5739–0.7930)	0.8239 (0.7432–0.9046)	0.7821 (0.7023–0.8619)	0.7427 (0.6438–0.8416)	0.7845 (0.7042–0.8648)
F1 score	0.7738 (0.6925–0.8551)	0.8817 (0.8229–0.9405)	0.8523 (0.7927–0.9119)	0.8224 (0.7516–0.8932)	0.8741 (0.8146–0.9336)
**Processed**	**Hemorrhages**	**Drusen**	**Hard exudates**	**Cotton wool spots**	**Retinal breaks**
AUC	0.9517 (0.9215–0.9819)	0.9378 (0.8771–0.9985)	0.9483 (0.9124–0.9842)	0.9815 (0.9617–1.0000)	0.9532 (0.9046–1.0000)
Sensitivity	0.9124 (0.8721–0.9527)	0.8638 (0.7645–0.9631)	0.9026 (0.8429–0.9623)	0.9142 (0.8357–0.9927)	0.8726 (0.7835–0.9617)
Specificity	0.9236 (0.8829–0.9643)	0.8735 (0.7732–0.9738)	0.8514 (0.7802–0.9226)	0.9923 (0.9725–1.0000)	0.9918 (0.9715–1.0000)
Precision	0.9817 (0.9728–1.0000)	0.9649 (0.9241–1.0000)	0.9451 (0.9043–0.9859)	1.0000 (1.0000–1.0000)	1.0000 (1.0000–1.0000)
Accuracy	0.9125 (0.8729–0.9521)	0.8629 (0.7623–0.9635)	0.8928 (0.8231–0.9625)	0.9426 (0.8732–1.0000)	0.9317 (0.8614–1.0000)
F1 score	0.9523 (0.9227–0.9819)	0.9124 (0.8329–0.9921)	0.9217 (0.8735–0.9709)	0.9529 (0.8932–1.0000)	0.9321 (0.8629–1.0000)

Values are the highest harmonic mean with the 95% confidence intervals.

### Evaluation and comparison of artifact removal methods

Preprocessing images through artifact removal can significantly improve lesion detection performance in deep learning systems. To assess the effectiveness of different artifact removal strategies, we conducted a comparative analysis involving three representative denoising techniques: (1) Center Crop. This method retains only the central elliptical region of the image, based on the fundus boundaries along the x- and y-axis diameters. While it effectively removes most border artifacts, it may inadvertently discard peripheral retinal regions, potentially omitting lesions located near the image margins. (2) Otsu Thresholding. This method refines the image by applying Otsu’s algorithm to distinguish and eliminate non-retinal background regions, providing a more precise segmentation of the valid area. (3) Attention-based Masking. Inspired by previous work (15), this strategy leverages attention maps generated by the model to identify and mask out low-attention regions, under the assumption that these areas are less informative or dominated by noise.

**FIGURE 5:**
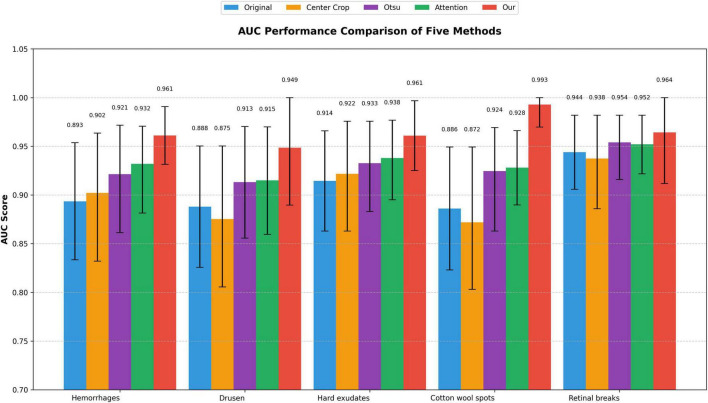
Areas under the curves (AUC) performance comparison of five methods.

Our results demonstrate that Module II, which integrates a more efficient and adaptive artifact removal mechanism in low cost, achieved the most significant improvement in lesion recognition accuracy across multiple categories. As illustrated in Figure 5, this approach outperformed the baseline and three competitors, suggesting that preserving critical lesion features while selectively removing noise-rich areas is essential for robust model performance.

### Evaluation of explainability

**FIGURE 6:**
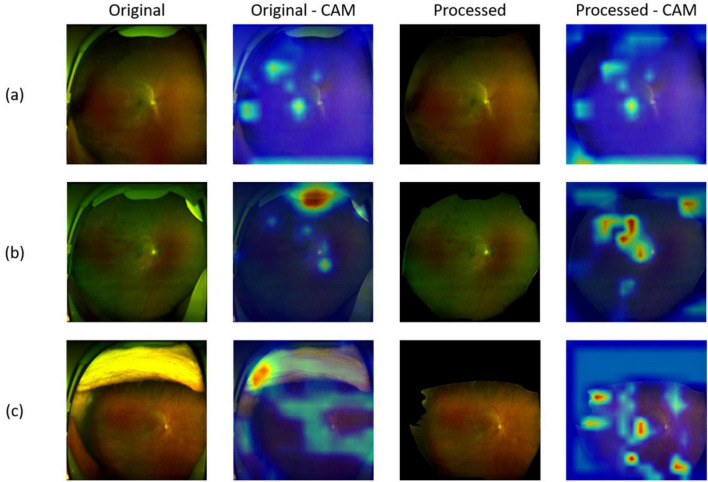
Representative images of original/processed UWF images and corresponding heatmaps generated using gradient-weighted class activation mapping (Grad-CAM). **(a)** Images and heatmaps with drusen; **(b)** Images and heatmaps with hemorrhage; **(c)** Images and heatmaps with both hemorrhage and drusen.

As we trained segmentation model as Module II to remove the artifacts, irrelevant information can be removed through this process. Figure 6 shows the images before processing (denoted as Original) and after processing (denoted as Processed). Moreover, to investigate the basis of the predictions output by the screening models and which part the models mainly cared about, we applied gradient-weighted class activation mapping (Grad-CAM) method on the corresponding fundus images from the positive individuals. Specifically, we extract the features from the last convolutional layer of ResNet-50, and then was used to calculate the probability distribution for the heatmap visualization. From Figures 6a–c, one UWF image with drusen, one UWF image with hemorrhage, and one image with both of hemorrhage and drusen were selected. It can be seen that, without artifacts removed, the heatmaps mainly focus on the eyelids. After artifacts were removed, more attention was paid on the lesions, which were clinically mainly cared about. The findings clearly validated Module II’s effectiveness, indicating that artifacts of UWF imaging can bring strong interference to the deep learning-based model training and prediction for a real-world screening.

## Discussion

In this study, we developed and evaluated a DL system using 5,759 UWF images for automated identification of five abnormal findings including hemorrhage, drusen, hard exudate, cotton-wool spot and retinal break. Consisting of all the three Modules (quality assessment module, artifact removal module and lesion recognition module), the DL system showed remarkable performance for the detection of hemorrhages, drusen, hard exudates, cotton wool spots and retinal breaks on the external validation dataset. This unprecedented success offered a promising way to accurately discriminate the five common retinal lesions in UWF images at one time, which might be particularly helpful in regular screening of diverse retinal diseases in general population. Besides, Our screening tool was trained with real-world non-mydriatic fundus images, which makes this tool especially suitable for patients with small pupil or mydriasis contraindication.

Sun et al. (28) developed a deep learning model based on ultra-widefield images that can identify eight fundus diseases, achieving good performance matching the capabilities of experienced fundus clinicians. In their study, they artificially excluded images with significant refractive media opacity or obvious treatment traces and images obscured by eyelids and eyelashes or the examiner’s finger, which might reduce the generalizability of the system. Antaki et al. (16) utilized AutoML model to differentiate retinal vein occlusion (RVO), retinitis pigmentosa (RP) and retinal detachment (RD) from normal fundi based on only one publicly available image data set, resulting in ordinary usability and generalizability of the model in spite of good performance. Cao et al. (29) established a four-level hierarchical eye diseases screening system with good performance in identifying up to 30 abnormalities and eye diseases based on UWF images. They designed a lesion atlas to recognize and describe retinal diseases on lesion level, thus increasing the expandability and interpretability particularly in multi-morbidity and comorbidity. However, their datasets consisted of images of patients with only target diseases and they also made image exclusion intentionally.

Different from former studies, our team did not undergo any filtering or cleaning on our data, and we emphasized quality assessing and artifact removing part, which makes our study unique. In Module II (artifact removal module), artifacts were replaced with black areas and we kept these areas as masks, providing processed-images for subsequent procedures. As a result, not only model performance improved (as shown in Tables 1, 2), but also Grad-CAM heatmaps showed that attention shifted from artifacts to lesions after processing, as shown in Figure 6. Similar to our Module I and Module II, Liu et al. (30) established a flow-cytometry-like model DeepFundus to evaluate color fundus images in terms of multidimensional quality properties including overall quality, clinical quality factors, affected retinal structures and refractive media opacity. And they integrated this image quality classifier into a disease detecting model as a prescreening tool to filter out poor-quality images, which efficiently enhanced the performance of the system detecting DR, AMD and optic disk edema. Another team also focused on the real-world clinical translation of the deep learning system (15). First of all, they not only kept images with multiple diseases in the same image, artifacts and borderline cases, but also totally “healthy” images, thus making the data unbalanced. Secondly, they evaluated their system on a challenging external dataset that included images with different preprocessing and images taken with various UWF imaging device models. As a result, their model showed lower AUCs than other published models. Yet they further proved that degraded performance resulted from challenging but realistic test data rather than inefficient approach. Apparently, their model trained for identifying images with disease and detecting seven retinal diseases would be more applicable under realistic conditions, despite the fact that they used only one public dataset. In future work, it is necessary for researchers to develop AI model based on multiple data sources to get close to reality. In addition, transfer learning (31), a technique aiming to transfer knowledge from one task to a different but related task is worth trying to enhance the generalizability of AI systems. All in all, to the best of our knowledge, our study was the first to develop a DL system consisting of three modules to recreate a whole screening process in clinical practice based on UWF images in three hospitals. And our results showed exactly how much influence artifacts would have on the DL system training process. We also compare our proposed methods with the existing methods specified for artifacts removal. Our findings highlight the critical role of artifact removal in improving deep learning-based lesion detection. While conventional strategies such as center cropping and thresholding offer straightforward solutions to reduce noise, they may also risk discarding peripheral or subtle pathological features. In contrast, our Module II approach demonstrates that a more targeted and adaptive denoising mechanism can better preserve clinically relevant information while suppressing irrelevant artifacts.

Although our DL system showed high accuracy in detecting MAF, there were still misclassifications. We found that most false-positive classifications came from regions with similar color or appearance. For example, some retinal pigment spots had a similar color to old retinal hemorrhages. And more than half of the false positives for drusen and hard exudates were due to white dots or hard reflections from the internal limiting membrane. When investigating the causes of false-negative classifications using the DL system without Module II, we observed that the majority were due to obscured MAF features caused by optical media opacity or imaging artifacts. Moreover, the imbalance in lesion prevalence may have also contributed to missed detections, particularly for underrepresented abnormalities. The incorporation of the artifact removal module significantly reduced these misclassifications by improving image clarity and feature visibility. However, a small number of false negatives remained, mainly attributable to MAFs that were too subtle or small to be identified by the system. Therefore, additional strategies to address class imbalance and enhance feature sensitivity are necessary to further minimize misclassifications and optimize the screening tool for clinical use.

This study has several limitations. First, as a retrospective study, our system was developed from images in only one medical center and was validated in only three Chinese hospital datasets. We need more datasets with more sources of variation and multiethnic clinic-based study assess its generalizability. In addition, it was hard to precisely detect subtle changes such as drusen and exudates around the posterior pole. Other examinations such as optical coherence tomography and more precise algorithms are needed in future work. Thirdly, although Optos can image up to 200° of the retina, some peripheral regions may not be observed, resulting in some peripheral lesions such as hemorrhages and retinal breaks missed by this screening system. Lastly, although our model is capable of producing multi-label predictions for a single image, this study only evaluated its performance on individual lesion types in isolation. As a result, we have not yet assessed its effectiveness in jointly recognizing multiple co-existing lesions. Further development and validation are needed to ensure robust multi-label diagnostic performance in complex clinical scenarios.

In conclusion, we developed a promising tool for screening real-world multiple abnormal findings based on non-mydriatic ultra-wide field fundus image with a high level of accuracy. Further multicenter validation is needed for a more comprehensive evaluation of the versatility of this DL system.

## Data Availability

The raw data supporting the conclusions of this article will be made available by the authors upon reasonable request.
