# Supplementary material for: Development and evaluation of a deep learning system for screening real-world multiple abnormal findings based on ultra-widefield fundus images
Source: Front Med (Lausanne). 2025 Jun 3;12:1584378. doi: 10.3389/fmed.2025.1584378 (PMC12170525; doi:10.3389/fmed.2025.1584378)
Supplement: Supplementary file 1 [file Table_1.docx]

**Supplementary Table 1. Characteristics of the training, internal validation, and external validation datasets.**

|  | XHH dataset | | | ZRH dataset | SBH dataset |
| --- | --- | --- | --- | --- | --- |
| Total no. of images | | 4521 | | 344 | 894 |
| Total no. of high-quality images | | 4289 | | 344 | 894 |
| No. of subjects | | 1504 | | 243 | 500 |
| Age, mean/range (years) | | 68.5/47-90 | | 67.1/43-81 | 45.8/25-80 |
| No. (%) of women | | 786(52.3) | | 138(56.8) | 268(53.6) |
| Ethnicity | | Han | | Han | Han |
| Camera model | | OPTOS Daytona P200T | | OPTOS Daytona P200T | OPTOS Daytona P200T |
|  | | Training dataset | Internal validation dataset | External validation dataset 1 | External validation dataset 2 |
| Hemorrhage | | 509 | 71 | 144 | 105 |
| Drusen | | 127 | 13 | 41 | 53 |
| Hard exudate | | 279 | 43 | 71 | 50 |
| Cotton wool spot | | 101 | 14 | 28 | 38 |
| Retinal break | | 92 | 16 | 26 | 13 |
| Normal | | 283 | 41 | 90 | 705 |
